# DPPN-SVM: Computational Identification of Mis-Localized Proteins in Cancers by Integrating Differential Gene Expressions With Dynamic Protein-Protein Interaction Networks

**DOI:** 10.3389/fgene.2020.600454

**Published:** 2020-10-23

**Authors:** Guang-Ping Li, Pu-Feng Du, Zi-Ang Shen, Hang-Yu Liu, Tao Luo

**Affiliations:** College of Intelligence and Computing, Tianjin University, Tianjin, China

**Keywords:** protein subcellular localization, differentially gene expression, protein-protein interactions, mis-localized proteins, diffusion kernel

## Abstract

Eukaryotic cells contain numerous components, which are known as subcellular compartments or subcellular organelles. Proteins must be sorted to proper subcellular compartments to carry out their molecular functions. Mis-localized proteins are related to various cancers. Identifying mis-localized proteins is important in understanding the pathology of cancers and in developing therapies. However, experimental methods, which are used to determine protein subcellular locations, are always costly and time-consuming. We tried to identify cancer-related mis-localized proteins in three different cancers using computational approaches. By integrating gene expression profiles and dynamic protein-protein interaction networks, we established DPPN-SVM (Dynamic Protein-Protein Network with Support Vector Machine), a predictive model using the SVM classifier with diffusion kernels. With this predictive model, we identified a number of mis-localized proteins. Since we introduced the dynamic protein-protein network, which has never been considered in existing works, our model is capable of identifying more mis-localized proteins than existing studies. As far as we know, this is the first study to incorporate dynamic protein-protein interaction network in identifying mis-localized proteins in cancers.

## Introduction

Eukaryotic cell is the most basic structural and functional unit of eukaryotic living creatures. Every cell contains numerous more basic components named subcellular compartments or subcellular organelles (Reece, 2015). According to the presence or absence of membranes, these subcellular organelles can be divided into two categories, the membrane bounded subcellular compartments and the non-membrane bounded subcellular structures (Perez-Ordonez et al., 2006). The membrane bounded subcellular compartments are those compartments surrounded by a single or double lipid layer membrane, such as mitochondria, nucleus and chloroplasts (in photosynthetic organisms). The non-membrane bounded subcellular structures, for example, the ribosomes, the cytoskeletons and the centrioles, are those structures without a membrane.

Proteins, which are translated in cytosol or rough ER (Endoplasmic Reticulum), must be transported to proper compartments during or after the translations to perform their biological functions (Mitra et al., 2006; Nyathi et al., 2013; Johnson et al., 2013). This process is known as the protein sorting process (Alberts et al., 2002). The subcellular organelles, where a protein performs its biological functions, are called the subcellular localization of the protein. A protein may have one or more than one subcellular localizations (Cheng et al., 2017). In complex disease conditions, some proteins may be sorted to incorrect subcellular locations, which results in abnormal intracellular behavior (Lee et al., 2008). For example, Zellweger syndrome is a rare congenital disorder characterized by the reduction or absence of functional peroxisomes in the cells of an individual (Brul et al., 1988). A study showed that many diseases such as Swyer syndrome, speech-language disorder, Alzheimer’s disease, kidney stones and Diamond-Blackfan anemia were all associated with mis-localized proteins (Hung and Link, 2011). Therefore, tracking alternative subcellular locations in different cellular conditions is important in understanding the pathology of complex diseases, like cancers.

With the help of automatic image processing and understanding technology, the first comprehensive human protein localization map was finally established (Uhlen et al., 2010; Thul et al., 2017). However, the experimental methods used to establish this kind of comprehensive localization map is still costly and time consuming (Horwitz and Johnson, 2017), which makes it difficult to establish this kind of localization map in different cellular conditions, such as disease conditions, drug perturbations and environmental stress conditions. Therefore, computational prediction approaches are still demanded in analyzing altered protein subcellular locations in different conditions.

During the last twenty years, hundreds of works have been done in predicting protein subcellular locations using various types of information at various levels of cellular structure in various species (Chou and Shen, 2006; Briesemeister et al., 2010; Mooney et al., 2011; Zhou et al., 2017; Cheng et al., 2017). For example, many works have been done in predicting protein subcellular locations using protein sequences and sequence related information (Chou and Shen, 2007; Du et al., 2011; Du and Xu, 2013). Most of these works rely on machine learning algorithms (Chou, 2011). Unfortunately, almost all existing studies, which focus on predicting protein subcellular locations, only predict subcellular locations for a given protein in only one condition (Liu and Hu, 2016).

This is because almost all existing studies of this kind utilize only the static information as the input data. For example, most of the existing methods tried to extract informative features from the primary sequence of proteins, while the mutations and the SNPs were not taken into considerations. For another example, some of the existing methods make use of the gene ontology annotations, as well as the functional domain composition of proteins (Zhou et al., 2017). There is still no distinguishable information that can be extracted from the gene ontology annotations or the functional domain compositions for different cellular conditions.

Several existing methods are designed to find the alternative protein subcellular locations in different cellular conditions. PROLocalizer makes use of sequence mutations to detect mis-localized protein in diseases (Laurila and Vihinen, 2009, 2011). Lee et al. integrated protein sequences, PPI (Protein-Protein Interaction) networks, and gene expression profiles to predict mis-localized proteins in glioma (Lee et al., 2008). Liu and Hu improved the Lee’s method to predict mis-localized protein in several types of cancers (Liu and Hu, 2016).

In these existing works, the information to distinguish different cellular conditions comes from two sources, one is the mutations and SNPs, while the other is the differential gene expressions. Although the gene mutation and SNP information is useful, it is not easy to utilize them in sequence based features. On the contrary, many gene expression datasets have been deposited in the NCBI GEO (Gene Expression Omnibus) database (Barrett et al., 2013), which have been proved to be useful if they are combined with the protein-protein interaction networks (Ideker and Krogan, 2012). Therefore, combining the gene expression profiles and the PPI network is a feasible way to explore mis-localized proteins in cancers, as well as other kinds of complex diseases.

Although state-of-the-arts methods, which applied gene expression profiles and PPI networks to predict mis-localized proteins in cancers, have achieved success in several specific types of cancers, it should be noted that these methods have two common issues.

First, all state-of-the-arts methods used identical PPI network structures in both the disease and non-disease conditions. This is the result of lacking PPI network data in specific disease conditions. However, if a protein is mis-localized in the disease condition, its interacting proteins must be changed, as the physical distances between the mis-localized protein and the other proteins are changed. Therefore, the topological structure of the PPI network in the disease condition must not be identical to the non-disease condition.

Second, as the topological structure of the PPI network should be changed in the disease condition, the difference of the topological structure of the PPI network should be utilized to predict mis-localized proteins.

In this work, we tried to solve the above two issues by building a model named DPPN-SVM (Dynamic Protein-Protein Network with Support Vector Machine). We made changes to the PPI network in the non-disease condition according to the changes of co-expression scores in disease condition to establish an adjusted PPI network in the disease condition. We applied the ECC (edge clustering coefficient), which has already been applied in predicting essential proteins and protein subcellular locations (Wang et al., 2012; Du and Wang, 2014), to extract the PPI network structure information. By training SVM classifiers with diffusion kernels (Kondor and Lafferty, 2002) on the PPI network, we can predict protein subcellular locations in different cellular conditions. We developed a mis-localization score, which describes how likely a protein will move to or leave from a specific subcellular location in a specific cellular condition. We hope this work may provide a better way in predicting mis-localized protein in various types of cancers.

## Materials and Methods

### PPI Network Construction

We downloaded our PPI data from the BioGRID database version 3.5.179 (Oughtred et al., 2019). To construct a high quality working dataset, we screened the raw PPI data strictly using the following criteria. (1) Only interactions between two human proteins were kept. (2) The interactions between two identical proteins were discarded, as this kind of interactions does not provide useful information for protein subcellular localizations. (3) Duplicate interaction records were reduced to unique interactions. (4) Only physical interactions were kept. All other types of interactions were removed. This is because the physical interactions implied that the two interactors have a very short physical distance, which contributes to protein subcellular location predictions. To achieve this, we kept only those interaction records with interaction type MI:0915 (physical association) or MI:0407 (direct interaction). After all above filtering procedures, we obtained 341088 interactions involving 23810 proteins.

### Subcellular Localization Annotations

We obtained reviewed human protein records from the UniProt database (UniProt Consortium, 2019), which include 20432 proteins. We employed the online ID mapping function of the UniProt database to convert the BioGRID protein IDs of every node in the PPI network to the UniProt database IDs. There are 16319 proteins in our PPI network, which can be mapped uniquely between the UniProt database and the BioGRID database. Although this covers just about 68% nodes in the PPI network, the number of interactions between these mapped proteins is 301366, which covers over 88% of all interactions.

After the mapping procedure, we transferred the GO (Gene Ontology) annotations in cellular component ontology category from the UniProt records to the BioGRID proteins. We chose the following 12 subcellular locations, including Cell cortex(GO:0005938), Cytosol(GO:0005829), Actin cytoskeleton(GO:0015629), Golgi apparatus(GO:0005794), Endoplasmic reticulum(GO:0005783), Nucleolus(GO:0005730), Peroxisome(GO:0005777), Mitochondrion(GO:0005739), Lysosome(GO:0005764), Centrosome(GO:0005813), Nucleus(GO:0005634), and Plasma membrane(GO:0005886). When the GO annotations were transferred from the Uniprot records to the BioGRID proteins, we choose to transfer only those GO terms with experimental evidences. This is achieved by choosing only those terms with evidence code IDA (Inferred from Direct Assay) or HDA (Inferred from High Throughput Direct Assay). We have 6461 BioGRID proteins that were experimentally annotated with at least one of the above 12 subcellular locations.

**FIGURE 1:**
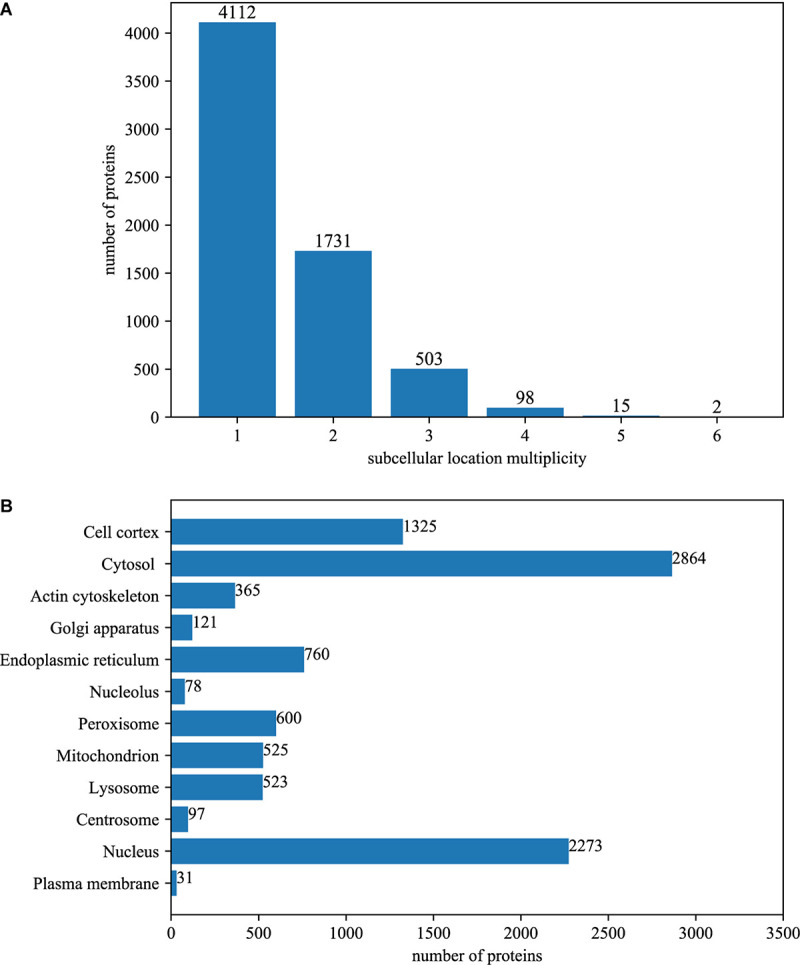
The summary of dataset. **(A)** The number of proteins with different number of subcellular locations. Among the 6461 annotated BioGRID proteins, there were 4112 proteins with only one subcellular location, 1731 proteins with two locations, 503 proteins with three locations, 98 proteins with four locations, 15 proteins with five locations and 2 proteins with six locations. The average multiplicity degree of the dataset was 1.48. **(B)** The number of locative proteins in different subcellular locations. There are 6461 proteins with experimentally annotated subcellular locations in the dataset. Because one protein may have more than one subcellular location, the number of locative proteins is 9562.

Among the 6461 annotated BioGRID proteins, there were 4112 proteins with only one subcellular location, 1731 proteins with two locations, 503 proteins with three locations, 98 proteins with four locations, 15 proteins with five locations and 2 proteins with six locations. The average multiplicity degree of the dataset was 1.48. The breakdown of the dataset for different location multiplicity is illustrated in Figure 1A.

### Virtual Locative Proteins

Since one protein may have more than one subcellular locations, it is necessary to introduce the virtual locative protein concept (Chou and Shen, 2006). In the view of machine learning, computational prediction of multiple subcellular locations for a single protein is a multi-label classification problem. Therefore, it should be converted to a single-label classification problem before it can be dealt with traditional machine-learning algorithms.

Every protein with *κ* (*κ* > 1) subcellular locations was split into *κ* virtual locative proteins. Each of the *κ* virtual locative proteins has one and only one of the *κ* subcellular locations. For example, if a protein *p*_*i*_ has two subcellular locations *l*_1_ and *l*_2_, we split the protein *p*_*i*_ into two different virtual proteins, located at *l*_1_ and *l*_2_, respectively.

The virtual locative proteins inherited the properties of the original real proteins, including all PPI connections and gene expression profiles. Since the virtual locative proteins have different subcellular locations, we assumed that there is no PPI between the virtual locative proteins that are generated from the same real protein.

The original 6461 proteins with experimentally annotated subcellular locations are split into 9562 virtual locative proteins, resulting in a multiplicity degree of 1.48. Therefore, the number of proteins that are mapped between UniProt and BioGRID increased to 19420, which is about 120% of the original. The number of PPI in the network increased to 601693, which is about 200% of the original. Figure 1B gives the breakdown of the dataset in the term of virtual locative proteins in different subcellular locations.

### Edge Clustering Coefficients

Edge clustering coefficient was originally developed in analyzing social networks (Radicchi et al., 2004). It has been introduced in identifying essential proteins (Wang et al., 2012), as well as in predicting protein subcellular locations (Du and Wang, 2014). Particularly, ECC has been proved to be an indicator of whether two interacting proteins tend to have common subcellular locations (Du and Wang, 2014). For a pair of interacting proteins, which can be noted as the *u*-th and the *v*-th proteins, the ECC can be defined as follows:

(1) $$\eta_{u,v}=\frac{z_{u,v}}{\min\left(d_{u}-1,d_{u}-1\right)},$$

where *η_*u*_*,*_*v*_* is the ECC between the *u*-th and the *v*-th proteins, *z*_*u*_,*_*v*_* the number of triangles that involve the edge between the *u*-th and the *v*-th proteins, and *d*_*u*_ and *d*_*v*_ the degree of the *u*-th and the *v*-th proteins, respectively.

The denominator in Eq (1) represents the possible most number of triangles that may involve the *u*-th and the *v*-th proteins. We set *η_*u*_*,*_*v*_* = 0 in the case that the denominator is degraded to zero.

### Diffusion Kernel Matrix

In order to apply machine learning techniques to graph-like structures, diffusion kernel was proposed to capture the long-range relationships between vertices induced by the local structure of a graph (Kondor and Lafferty, 2002). The diffusion kernels provide means to incorporate all neighbors of proteins in the network (Lee et al., 2006).

Let *G* be a simple graph. Its Laplacian matrix can be defined as:

(2) L=D-A,

where **A** is the adjacency matrix of the graph, and **D** the degree matrix. The matrix **D** can be defined as:

(3) $$D=\left\{d_{i,j}\right\}=\left\{ \begin{array}{cc} d_{i} & i=j\\ 0 & O\,t\,h\,e\,r\,w\,i\,s\,e \end{array}\right.,$$

where *d*_*i*_ is the degree of the *i*-th vertex in the graph. The diffusion kernel matrix **K**(*τ*) is given by:

(4) K⁢(τ)=exp⁡(-τ⁢L),

where *τ* is a constant parameter, exp() the matrix exponential function. It can be easily shown that the **K**(*τ*) is a valid kernel function.

### Co-expression Network Construction

Three cancer-related gene expression profile datasets were obtained from the NCBI GEO database. These datasets are from studies on acute myeloid leukemia, breast cancer and hepatitis carcinoma, respectively. The datasets include GSE9476 (myeloid leukemia, 25 cases and 38 controls), GSE27567 (breast cancer, 51 cases and 31 controls) and GSE121248 (hepatitis carcinoma, 70 cases and 37 controls). All gene expression datasets were retrieved using the Affymetrix platforms (Dalma-Weiszhausz et al., 2006). We used the “simpleaffy” package in the Bioconductor to perform quality controls (Wilson and Miller, 2005). For each dataset, the following filtering steps were carried out. (1) The samples with scale factors larger than 3 were removed. (2) The samples with 3′ to 5′ ratios for β-actin less than 3 were kept. (3) The samples with 3′ to 5′ ratios for GAPDH (Glyceraldehyde 3-phosphate dehydrogenase) less than 1.25 were kept. We also checked the RLE (relative log expression) and NUSE (normalized unscaled standard errors) of samples. Samples with significant different RLE or NUSE values to other samples were removed. The case and control samples in each dataset were grouped, respectively. The MAS5 algorithm (Pepper et al., 2007) were applied to generate expression values for every sample. We applied the affymetrix templates and annotation packages in Bioconductor to map the gene expression values to UniProt proteins. In case of a many-to-one mapping, we used the mean value as the final expression value for proteins.

Let *x*_*i*_,*_*u*_* be the *u*-th protein expression values of the *i*-th sample, *n* the number of samples in a group. We define the sample-wise centered expression vector **X***_*u*_* as follows:

(5) $$X_{u}={\left[\begin{array}{cccc} x_{1,u}-a_{u} & x_{2,u}-a_{u} & \cdots & x_{n,u}-a_{u} \end{array}\right]}^{T},$$

where *T* is the transpose operator for matrix, and

(6) $$a_{u}=\frac{1}{n}\,\sum_{i-1}^{n}x_{i,u}.$$

We now defined the pair-wise PCC (Pearson Correlation Coefficient) between the *u*-th protein and the *v*-th protein as the follows:

(7) $$\rho_{u,v}=\frac{X_{u}^{T}\,X_{v}}{\sqrt{X_{u}^{T}\,X_{u}}\,\sqrt{X_{v}^{T}\,X_{v}}},$$

where *ρ_*u*_*,*_*v*_* is the PCC between the *u*-th and the *v*-th proteins.

The PCC was used to quantify the coherent extent of two proteins in terms of gene expressions. Regardless to whether two proteins have physical interactions, their PCC was calculated as above.

### Disease-Related Mis-Localized Protein Identification

Given a specific disease status *θ*, we term the case sample set as *θ*_1_, while the control sample set as *θ*_0_.

We can compute the PCC for all pairs of proteins as Eq(7) using only the samples in *θ*_0_. The PCC between the *u*-th and the *v*-th proteins in non-disease states can be noted as *ρ_*u*_*,*_*v*_*(*θ*_0_). Similarly, we can compute the ECC for each interaction as Eq(1). The ECC between the *u*-th and the *v*-th proteins in non-disease states can be noted as *η_*u*_*,*_*v*_*(*θ*_0_).

Let **A**(*θ*_0_) be the adjacency matrix of the PPI network in non-disease states, which can be defined as follows:

(8) $$A\,\left(\theta_{0}\right)=\left\{a_{u,v}\,\left(\theta_{0}\right)\right\}=\left\{ \begin{array}{cc} \rho_{u,v}\,\left(\theta_{0}\right)\,\eta_{u,v}\,\left(\theta_{0}\right) & T\,h\,e\,\text{u-th}\,a\,n\,d\,\text{v-th}\,p\,r\,o\,t\,e\,i\,n\,a\,r\,e\,i\,n\,t\,e\,r\,a\,c\,t\,i\,n\,g\\ 0 & o\,t\,h\,e\,r\,w\,i\,s\,e \end{array}\right..$$

The Laplacian matrix in non-disease state can be defined as:

(9) $$L\,\left(\theta_{0}\right)=D\,\left(\theta_{0}\right)-A\,\left(\theta_{0}\right),$$

where **D**(*θ*_0_) is the degree matrix that is computed using Eq(3).

With **L**(*θ*_0_), we can create the diffusion kernel matrix **K**(*τ*,*θ*_0_) using Eq(4). This kernel matrix is used in an SVM model to predict protein subcellular locations in the non-disease state. Since we took the multi-label scenario into the consideration, we employed the libSVM package (Chang and Lin, 2011) to derive the probability that each locative protein localized to each subcellular locations.

Let *p*_*u*_,*_*k*_*(*θ*_0_) be the probability score that the *u*-th protein localize to the *k*-th subcellular location. The libSVM package ensures that

(10) $$\sum_{k=1}^{m}p_{u,k}\,\left(\theta_{0}\right)=1,$$

where *m* is the number of all possible subcellular locations.

Due to the imbalanced dataset, the ranges of *p*_*u*_,*_*k*_*(*θ*_0_) of different subcellular locations varies a lot. Therefore, we defined the following adjusted probability score, *q*_*u*_,*_*k*_*(*θ*_0_), which is for the *u*-th protein and the *k*-th subcellular location:

(11) $$q_{u,k}\,\left(\theta_{0}\right)=\frac{{\hat{p}}_{u,k}\,\left(\theta_{0}\right)}{\sum_{k=1}^{m}{\hat{p}}_{u,k}\,\left(\theta_{0}\right)},$$

where

(12) $${\hat{p}}_{u,k}\,\left(\theta_{0}\right)=\frac{p_{u,k}\,\left(\theta_{0}\right)-\min_{u}p_{u,k}\,\left(\theta_{0}\right)}{\max_{u}p_{u,k}\,\left(\theta_{0}\right)-\min_{u}p_{u,k}\,\left(\theta_{0}\right)}.$$

With all above definitions, the *u*-th protein localize to the *k*-th subcellular location if the following condition is satisfied:

(13) $$q_{u,k}\,\left(\theta_{0}\right)\geq \max_{k}q_{u,k}\,\left(\theta_{0}\right)-\alpha \,\left(\max_{k}q_{u,k}\,\left(\theta_{0}\right)-\min_{k}q_{u,k}\,\left(\theta_{0}\right)\right),$$

where α is a real number parameter between 0 and 1. The subcellular locations, which are predicted for the *u*-th protein using Eq(13), can be denoted as a set **S***_*u*_*(*θ*_0_).

For the disease state, all above computation can be performed on *θ*_1_. However, to amplify the differences between disease and non-disease status, we altered the topology of the PPI network before all computations in disease status. This is different to all existing works in predicting mis-localized proteins in diseases.

For the *u*-th protein and the *v*-th protein, we first compute the PCC in *θ*_1_, which can be noted as *ρ_*u*_*,*_*v*_*(*θ*_1_). We define the disease status difference of PCC as follows:

(14) $$h_{u,v}=\rho_{u,v}\,\left(\theta_{1}\right)-\rho_{u,v}\,\left(\theta_{0}\right).$$

We define two threshold parameters as follows:

(15) $$t_{+}=h+3\,\sigma ,\text{and}$$

(16) $$t_{-}=h-3\,\sigma ,$$

where *h* is the average value of all *h*_*u*_,*_*v*_*, and *σ* the standard deviation of all *h*_*u*_,*_*v*_*.

If the *u*-th protein and the *v*-th protein are two interacting proteins in non-disease status, the interaction would be removed, if *h*_*u*_,*_*v*_* < *t*_–_ is satisfied. Similarly, if the *u*-th protein and the *v*-th protein are two non-interacting proteins in non-disease status, the interaction between them should be established, if *h*_*u*_,*_*v*_* > *t*_+_ is satisfied.

After altering the topology of the PPI network as above, we compute the **S***_*u*_*(*θ*_1_) according to the Eq(8) to Eq(13) using the updated PPI network and gene expression samples in *θ*_1_. It should be noted that the *η_*u*_*,*_*v*_*(*θ*_1_) may be different to *η_*u*_*,*_*v*_*(*θ*_0_), as the topology of the PPI network is altered in the disease state.

By comparing the **S***_*u*_*(*θ*_1_) and **S***_*u*_*(*θ*_0_), we can identify whether the subcellular locations of the *u*-th protein were altered in the disease state. However, this method cannot quantify how likely a protein would be mis-localized in the disease state. Therefore, we developed the following method to quantify the mis-localized proteins, which we termed as the mis-localization scores.

For each disease, we compute the differences of adjusted probability scores between the disease and non-disease states. The mis-localization score of the *u*-th protein in the *k*-th subcellular location of disease *θ* can be defined as follows:

(17) $$\varphi_{u,k}\,\left(\theta \right)=\frac{q_{u,k}\,\left(\theta_{1}\right)-q_{u,k}\,\left(\theta_{0}\right)}{q_{u,k}\,\left(\theta_{0}\right)}.$$

The *φ_*u*_*,*_*k*_*(*θ*) indicates the extent that the *u*-th protein would localize to or move from the *k*-th subcellular location. For each protein, we define the following two boundaries:

(18) $$\sup\left[\varphi_{u}\,\left(\theta \right)\right]=\max_{k}\varphi_{u,k}\,\left(\theta \right),\mathrm{and}$$

(19) $$\inf\left[\varphi_{u}\,\left(\theta \right)\right]=\min_{k}\varphi_{u,k}\,\left(\theta \right)$$

We sorted the proteins according to the sup[*φ_*u*_*(*θ*)] and inf[*φ_*u*_*(*θ*)] in descending and ascending orders, respectively. The top-ranked proteins within a fixed proportion of the entire list are considered as mis-localized proteins. The proportion is fixed as 0.1% in this work.

### Performance Evaluation Methods

In this study, we used 10-fold cross-validation to evaluate the prediction performance of our method in the non-disease state. Four statistics, including aiming (AIM), coverage (CVR), multi-label accuracy (mlACC), absolute-true rate (ATR) were applied to measure the prediction performances (Jiao and Du, 2016). These statistics are defined as follows:

(20) $$A\,I\,M=\frac{1}{b}\,\sum_{u=1}^{b}\left|\frac{S_{u}\,\left(\theta_{0}\right)\,⋂S_{u}}{\left|S_{u}\,\left(\theta_{0}\right)\right|}\right|,$$

(21) $$C\,V\,R=\frac{1}{b}\,\sum_{u=1}^{b}\left|\frac{S_{u}\,\left(\theta_{0}\right)\,⋂S_{u}}{\left|S_{u}\right|}\right|,$$

(22) $$\text{mlACC}=\frac{1}{\text{b}}\,\sum_{u=1}^{\text{b}}\left|\frac{{\text{S}}_{\text{u}}\,\left(\theta_{0}\right)\,⋂{\text{S}}_{\text{u}}}{{\text{S}}_{\text{u}}\,\left(\theta_{0}\right)\,⋃{\text{S}}_{\text{u}}}\right|,\text{and}$$

(23) $$A\,T\,R=\frac{1}{b}\,\sum_{u=1}^{b}\delta \,\left[S_{u}\,\left(\theta_{0}\right),S_{u}\right],$$

where **S***_*u*_*(*θ*_0_) is the set of predicted protein subcellular locations of the *u*-th protein in the non-disease state, **S***_*u*_* the set of experimental protein subcellular locations, *b* the number of proteins, | .| the cardinal operator in set theory, and

(24) $$\delta \,\left[S_{u}\,\left(\theta_{0}\right),S_{u}\right]=\left\{ \begin{array}{cc} 1 & S_{u}\,\left(\theta_{0}\right)=S_{u}\\ 0 & o\,t\,h\,e\,r\,w\,i\,s\,e \end{array}\right..$$

Since we have introduced the virtual locative proteins in our work, we also applied single-label performance measures. Five statistics, including sensitivity (Sen), specificity (Spe), virtual-locative accuracy (vlAcc), positive-predictive value (PPV) and Matthew’s Correlation Coefficients (MCC) are applied in our work. These statistics can be defined as follows:

(25) $$S\,e\,n=\frac{T\,P}{T\,P+F\,N},$$

(26) $$S\,p\,e=\frac{T\,N}{T\,N+F\,P},$$

(27) $$P\,P\,V=\frac{T\,P}{T\,P+F\,P},$$

(28) $$\text{vlAcc}=\frac{\mathrm{TP}+\mathrm{TN}}{\mathrm{TP}+\mathrm{TN}+\mathrm{FP}+\mathrm{FN}},\text{and}$$

(29) $$M\,C\,C=\frac{T\,P\,T\,N-F\,P\,F\,N}{\sqrt{\left(T\,P+F\,P\right)\,\left(T\,P+F\,N\right)\,\left(T\,N+F\,P\right)\,\left(T\,N+F\,N\right)}},$$

where *TP*, *TN*, *FP* and *FN* are the numbers of true positives, true negatives, false positives, and false negatives in the cross-validation, respectively.

### Parameter Calibrations

We used a grid search strategy to find the parameter combination of τ and α that optimize the 10-fold cross validation performances in the non-disease state. The parameter *τ* in computing the diffusion kernel was searched from 0.1 to 2.0 with step 0.1. The parameter α in Eq(13) was searched from 0.1 to 0.3 with a step of 0.1. Supplementary Figure 1 showed the global MCC score under different parameters. We chose the parameter values *τ* = 1.1 and α = 0.3 in our works.

## Results and Discussion

### Prediction Performance Analysis in the Non-disease State

We used 10-fold cross-validation to evaluate the prediction performances in non-disease state. It should be noted that our method is designed to find out the alteration of protein subcellular locations, rather than the exact subcellular locations in non-disease state. Therefore, we choose to compare our method to Liu and Hu’s method (Liu and Hu, 2016). Since we applied virtual locative protein concept in our work, while Liu and Hu employed the top-k accuracy performance measure, it is difficult to perform an exact apple-to-apple orange-to-orange comparison. However, we managed to compare the global sensitivity of our work to the top-1 accuracy of Liu and Hu’s work. As our performance value was obtained by using 10-fold cross-validation, this gives some advantage to Liu and Hu’s work. Our global sensitivity is 0.556, while the top-1 accuracy of Liu and Hu’s work is 0.364. Although both values are not high enough in the general protein subcellular location predictions, we still achieved a comparable or little higher performance. Other global performance measure in terms of virtual locative proteins are a specificity of 0.899, a PPV of 0.437, an accuracy of 0.857 and an MCC of 0.412.

To make further performance assessment, we choose to compare the multi-label performance of our method to the Hum-mPLoc 3.0, which was developed by using gene ontology information. Since our method does not rely on the gene ontology annotations, which has been proved to have superior performances in predicting protein subcellular locations, it should be noted that the Hum-mPLoc 3.0 (Zhou et al., 2017) has intrinsic performance advantages.

Since Hum-mPLoc 3.0 does not use identical subcellular locations annotations as our method, we choose to compare the overlapped locations. To achieve a fair enough comparison, we compose a testing dataset of 3842 proteins. All these proteins are with at least one overlapped subcellular location. This testing dataset was fed into the Hum-mPLoc 3.0 and our method in non-disease state. The overall multi-label performances were compared in Table 1. It can be seen that our method has better performance in terms of aiming, coverage, accuracy and absolute true rate. This is an expectable result, as our method incorporates PPI information and gene expression profiles.

**TABLE 1:** Performance comparison in non-disease state.

Measures^a^	Our method	Hum-mPLoc 3.0
AIM	72.00%	68.10%
CVR	69.50%	65.10%
mlACC	68.60%	65.00%
ATR	64.30%	61.80%

*^*a*^ All performance measures are defined in Eq (20), (21), (22), and (23).*

### Discovery of Potentially Mis-Localized Proteins in Cancers

We applied our method on three different type of cancers, including leukemia, breast cancer and hepatitis carcinoma. Table 2 gives a list of representative mis-localized proteins in these cancer cells. For each disease, we listed the top six (0.1% of the entire list) proteins, which are most likely to mis-localize to an abnormal location, and the top six proteins, which are most likely to mis-localize from their normal locations. The corresponding location, the mis-localization score and the score rank can also be found in Table 2. In addition, we listed some highly ranked proteins that has been reported to be related to cancers by other literatures.

**TABLE 2:** Representative prediction of mis-localized proteins.

Disorder	Uniprot ID	Mis-localizations^a^	Rank^b^
Leukemia	MAGA3_HUMAN	+Cell cortex (+Inf)	1
	F217B_HUMAN	+Peroxisome (+Inf)	2
	EI24_HUMAN [41]	+Mitochondrion (+3349.02%)	3
	ROP1A_HUMAN	+Peroxisome(+3086.82%)	4
	THYN1_HUMAN	+Nucleus (+2461.70%)	5
	CLGN_HUMAN	+Nucleus (+2425.50%)	6
	SETBP_HUMAN [40]	+Endoplasmic reticulum (+658.04%)	30
	TF2L1_HUMAN	−Nucleus (−99.57%)	1
	UPP1_HUMAN	−Cell cortex (−97.49%)	2
	AL1A1_HUMAN	−Peroxisome (−96.63%)	3
	ABCA1_HUMAN	−Lysosome (−95.71%)	4
	PARP4_HUMAN	−Lysosome (−94.87%)	5
	AL7A1_HUMAN	−Peroxisome (−93.09%)	6
	SETBP_HUMAN [40]	−Nucleus (−83.94%)	28
	EI24_HUMAN [41]	−Endoplasmic reticulum(−50.22%)	348
Breast cancer	TM258_HUMAN	+Peroxisome (+Inf)	1
	KCNKI_HUMAN	+Mitochondrion (+Inf)	2
	MARC2_HUMAN	+Cell cortex (+Inf)	3
	HEBP2_HUMAN	+Lysosome (+Inf)	4
	SIT1_HUMAN	+Mitochondrion (+13310.16%)	5
	PIM3_HUMAN	+Lysosome (+9723.01%)	6
	PD1L1_HUMAN [42]	+Nucleolus (+290.65%)	242
	INGR2_HUMAN [43]	+Mitochondrion (+184.50%)	437
	VGFR3_HUMAN [42]	+Nucleolus (+125.16%)	755
	ANO4_HUMAN	−Nucleus (−98.91%)	1
	ABCA1_HUMAN	−Lysosome (−98.78%)	2
	NDUB7_HUMAN	−Mitochondrion (−98.35%)	3
	TM127_HUMAN	−Plasma membrane (−98.25%)	4
	RUBIC_HUMAN	−Endoplasmic reticulum (−96.45%)	5
	TRIM4_HUMAN	−Nucleus (−96.45%)	6
	INGR2_HUMAN [43]	−Plasma membrane (−63.74%)	595
Hepatitis carcinoma	TBCA_HUMAN	+Cell cortex (+Inf)	1
	F217B_HUMAN	+Peroxisome (+Inf)	2
	HKDC1_HUMAN	+Nucleus (+65006.48%)	3
	SYAC_HUMAN	+Peroxisome (+10652.77%)	4
	RFWD3_HUMAN	+Lysosome (+10599.05%)	5
	ABCA1_HUMAN [10]	+Lysosome (+8115.45%)	6
	S10AB_HUMAN [44]	+Peroxisome (+6868.17%)	12
	FOXP1_HUMAN [10]	+Peroxisome (+612.39%)	478
	RM14_HUMAN	−Cell cortex (−99.99%)	1
	RM47_HUMAN	−Cell cortex (−99.99%)	2
	RT30_HUMAN	−Cell cortex (−99.99%)	3
	DUS11_HUMAN	−Cell cortex (−99.99%)	4
	CLGN_HUMAN	−Cell cortex (−99.99%)	5
	RM01_HUMAN	−Cell cortex (−99.99%)	6
	ABCA1_HUMAN [10]	−Cell cortex (−99.28%)	249

*^*a*^ The mis-localization score is marked after the altered location. The “+” prefix indicates this is a new subcellular location in disease state. The “−” prefix indicates this non-disease subcellular location is lost in the disease state. The “Inf” indicates a positive infinity value, which is produced by the zero original localization probability. ^*b*^ The score ranks are sorted using the boundary values in Eq(18) and Eq(19). The mis-localization scores with value of −100% does not participate in the ranking, as it does not necessarily indicate a completely loss of a subcellular location, but just a bias of available data.*

A comprehensive list of all proteins with the mis-localization scores can be found in supplementary data. In supplementary data, Supplementary Tables 1–3 contain the comprehensive lists of the localization scores under different state and mis-localization scores of three diseases with all locations, one table per disease. Supplementary Tables 4–15 are comprehensive lists of sorted mis-localization scores for hepatitis in different locations, one table per location. Supplementary Tables 16–27 are comprehensive lists of sorted mis-localization scores for leukemia in different locations. Supplementary Tables 28–39 are comprehensive lists of sorted mis-localization scores for breast cancer in different locations. Supplementary Tables 40–42 are comprehensive lists of sorted maximum mis-localization scores, one table per disease. Supplementary Tables 43–45 are comprehensive lists of sorted minimum mis-localization scores, one table per disease.

#### Leukemia

In acute myeloid leukemia, we used 25 cases and 38 controls. Our prediction showed that protein SETBP1 mis-localized to ER in cancer cells, as its localization score in ER increased from 0.083 to 0.633 with a mis-localization score +658.04%, while its localization score in nucleus dropped from 0.226 to 0.036 with the mis-localization score −83.94%. A recent study have suggested a direct involvement of SETBP1 in leukemia development (Oakley et al., 2012). We predicted that EI24 mis-localized from ER in cancer cells, as its localization score drops from 0.94 to 0.468 with a mis-localization score −50.22%, while Zhao et al. (2005) found that EI24/PIG8 was an ER-localized Bcl2-binding protein, which was highly mutated in aggressive breast cancers.

#### Breast Cancer

For breast cancer, we used 51 cases and 31 controls. We made a prediction that the protein B7H1 mis-localized from plasma membrane and to nucleus, as its localization score in plasma membrane dropped from 0.243 to 0.105 with a mis-localization score −56.76%, while its localization score in nucleolus increased from 0.023 to 0.092 with the mis-localization score +290.65%. This consists with the record in literature (Wang and Li, 2014). Our method also reported that the protein VEGFR3 mis-localized from plasma membrane and to cell nucleus, as its localization score in cell nucleus increased from 0.047 to 0.106 (with the mis-localization score +125.16%). This also consists with the record in literature (Wang and Li, 2014). The protein IFNgR2 was annotated with location ER and Golgi apparatus in the Uniprot database. We predicted that IFNgR2 mis-localized from plasma membrane to mitochondria in cancer cells, as its localization score in plasma membrane drop from 0.214 to 0.078, with a mis-localization score −63.74%, while the localization score in mitochondria increased from 0.136 to 0.388 (with a mis-localization score +184.5%). It was reported that the IFNgR2 molecules can be mainly detected in mitochondria in cancer cells (Ngo et al., 2012).

#### Hepatitis Carcinoma

For hepatitis carcinoma, we used 70 cases and 37 controls. The protein S100A11 was reported to have very weak nuclear expression in adenocarcinomas (Rehman et al., 2004), while our method reported that it mis-localized to peroxisome, as its localization score in peroxisome increased from 0.014 to 1.0 (with a mis-localization score +6868.17%). We predicted that FOXP mis-localized to peroxisome, as its localization score in peroxisome increased from 0.003 to 0.019 with mis-localization score 612.39%. It has been reported that FOXP would lose its nuclear localization in cancers (Hung and Link, 2011). ABCA1 was reported to mis-localize from plasma membrane to lysosome in cancers (Hung and Link, 2011). Our method reported the same result, as the localization score in plasma membrane dropped from 0.162 to 0.004 with mis-localization score −99.28%), and lysosome from 0.012 to 0.972 (with a mis-localization score +8115.45%).

### Potential Results Validation

Using our method, we identified some proteins that may mis-localize from or to a specific location. Some of them have been verified by existing studies. But most of the predicted proteins have not been verified. Due to our limited resources, we cannot perform experimental validations. This may be considered as a future work. It should also be noted that, there is still no database for mis-localized proteins. The information regarding the mis-localized proteins is still scattered in many literatures. Establishing such kind of database is a valuable yet impacting work, which is also in our consideration as a future work in this research topic. Since mis-localized proteins are of great significance on revealing the mechanism of diseases, we believe that it is valuable to establish a database to summarize and store relevant discoveries in future.

## Conclusion

Computational prediction of proteins subcellular locations has been studied for over twenty years. However, computationally detecting disease-related mis-localized proteins was rarely discussed. By integrating gene expression profiles and protein-protein interaction networks, we developed a computational approach, DPPN-SVM, to detect mis-localized proteins in various cancers. The results indicated that our method can successfully identify cancer-related or mis-localized proteins that has been reported in various literatures. Comparing to existing studies, our method not only provide a comparable or better prediction performance in non-disease state, but also further amplify the differentially expressed gene information by introducing the dynamic PPI network and the SVM classifiers with diffusion kernels. The prediction results of our method provide candidate proteins as spatial cancer markers, while the method of our work gives a new way to explore the spatial distribution of proteins within a cell.

## Data Availability Statement

Publicly available datasets were analyzed in this study. This data can be found here: https://github.com/brown-2/mis_localization.

## Author Contributions

G-PL collected data, process the data, implement the algorithm, performed most of the experiments, and analyzed the results. P-FD designed and directed the study, proposed the algorithm, analyzed the results, and wrote the manuscript. Z-AS and H-YL performed part of the experiments. TL analyzed the results, and participated in writing the manuscript. All authors contributed to the article and approved the submitted version.

## Conflict of Interest

The authors declare that the research was conducted in the absence of any commercial or financial relationships that could be construed as a potential conflict of interest.
