# Supplementary material for: DPPN-SVM: Computational Identification of Mis-Localized Proteins in Cancers by Integrating Differential Gene Expressions With Dynamic Protein-Protein Interaction Networks
Source: Front Genet. 2020 Oct 23;11:600454. doi: 10.3389/fgene.2020.600454 (PMC7644922; doi:10.3389/fgene.2020.600454)
Supplement: Supplementary file 2 [file Presentation_1.PPTX]

## Slide 1
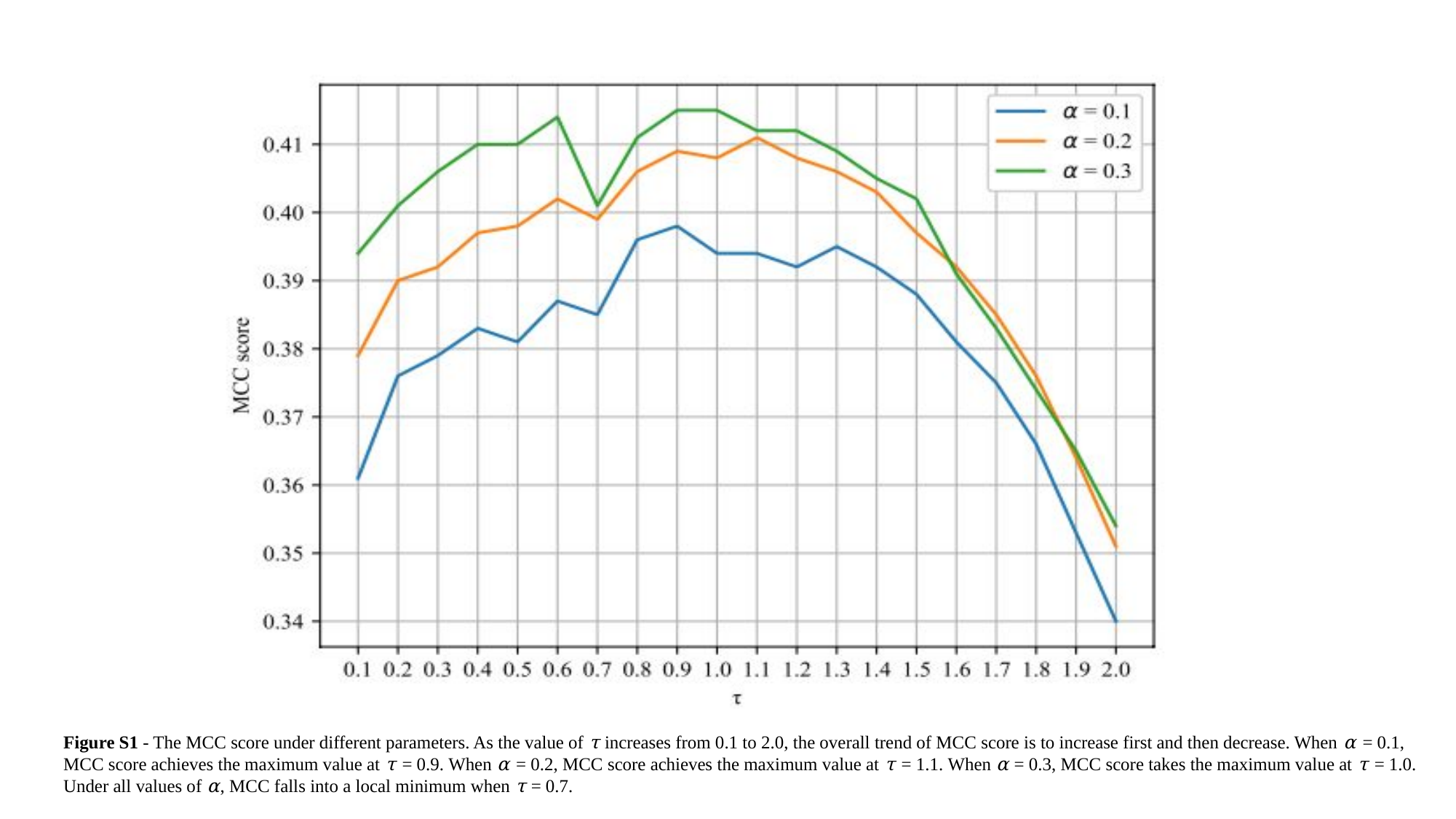

Figure S1 - The MCC score under different parameters. As the value of τ increases from 0.1 to 2.0, the overall trend of MCC score is to increase first and then decrease. When α = 0.1, MCC score achieves the maximum value at τ = 0.9. When α = 0.2, MCC score achieves the maximum value at τ = 1.1. When α = 0.3, MCC score takes the maximum value at τ = 1.0. Under all values of α, MCC falls into a local minimum when τ = 0.7.
